# Multienzyme Immobilized Polymeric Membrane Reactor for the Transformation of a Lignin Model Compound

**DOI:** 10.3390/polym10040463

**Published:** 2018-04-23

**Authors:** Rupam Sarma, Md. Saiful Islam, Mark P. Running, Dibakar Bhattacharyya

**Affiliations:** 1 Department of Chemical and Materials Engineering, University of Kentucky, Lexington, KY 40506, USA; r.sarma@uky.edu (R.S.); md.saifulislam@uky.edu (M.S.I.); 2 Department of Biology, University of Louisville, Louisville, KY 40292, USA; mark.running@louisville.edu

**Keywords:** polymer membrane reactor, multienzyme, lignin, layer-by-layer assembly

## Abstract

We have developed an integrated, multienzyme functionalized membrane reactor for bioconversion of a lignin model compound involving enzymatic catalysis. The membrane bioreactors were fabricated through the layer-by-layer assembly approach to immobilize three different enzymes (glucose oxidase, peroxidase and laccase) into pH-responsive membranes. This novel membrane reactor couples the in situ generation of hydrogen peroxide (by glucose oxidase) to oxidative conversion of a lignin model compound, guaiacylglycerol-β-guaiacyl ether (GGE). Preliminary investigation of the efficacy of these functional membranes towards GGE degradation is demonstrated under convective flow mode. Over 90% of the initial feed could be degraded with the multienzyme immobilized membranes at a residence time of approximately 22 s. GGE conversion product analysis revealed the formation of oligomeric oxidation products upon reaction with peroxidase, which may be a potential hazard to membrane bioreactors. These oxidation products could further be degraded by laccase enzymes in the multienzymatic membranes, explaining the potential of multi enzyme membrane reactors. The multienzyme incorporated membrane reactors were active for more than 30 days of storage time at 4 °C. During this time span, repetitive use of the membrane reactor was demonstrated involving 5–6 h of operation time for each cycle. The membrane reactor displayed encouraging performance, losing only 12% of its initial activity after multiple cycles of operation.

## 1. Introduction

Integration of enzymatic catalysis with membrane technology has attracted growing attention to facilitate functionalized membranes as bioreactors [1,2,3,4]. Synthetic membranes provide a versatile platform for immobilization of bio-catalysts (enzymes), thereby overcoming the inadequacies of soluble enzymes such as instability, difficult recovery, trouble in handling and non-reusability [5,6]. Enzymatic reactions, on the other hand, encourage emerging technologies incorporating multienzyme systems, making catalytic strategies operative and sophisticated [7,8,9]. Immobilized multienzymatic systems that exploit the selectivity of biocatalysts have been developed from time to time [10,11]. In this respect, functionalized membranes with porous support and functional polymer matrices may be an ideal platform for multiple enzyme immobilization and thus aid in developing bioreactors for enzymatic reactions [12,13].

Lignocellulosic feedstocks have received continuous attention as renewable biomass for the generation of biofuels and fine chemicals [14]. In particular, the highly abundant polymer, lignin, deserves more attention than only getting used for low-value applications such as low-grade fuel [15]. Plenty of research has been conducted that reports ways to exploit the prospective of lignin as a resource for value-added chemicals [14,16,17]. However, practical utility is far away owing to the challenges involved during lignin depolymerization. Most of the methods reported for lignin valorization, such as pyrolysis, catalytic oxidation and/or hydrolysis under supercritical conditions, etc., are either energy-consuming or environmentally unfavorable [18]. In nature, lignin is degraded by a pool of extracellular ligninolytic enzymes such as peroxidases and laccases over a period of many years [19]. One of the novel approaches to mimic natural ways of lignin depolymerization is involving multienzymatic reactions. Membrane-based multienzyme systems can be constructed by carrying out sequential deposition onto the membrane pores. The layer-by-layer (LbL) adsorption technique is a general and versatile tool for the controlled fabrication of surfaces and pores by the consecutive deposition of alternatively-charged polyelectrolytes [20,21,22]. Efforts have been made to fabricate multienzyme surfaces through LbL techniques for bioprocessing applications [23,24,25,26,27].

**Scheme 1:**
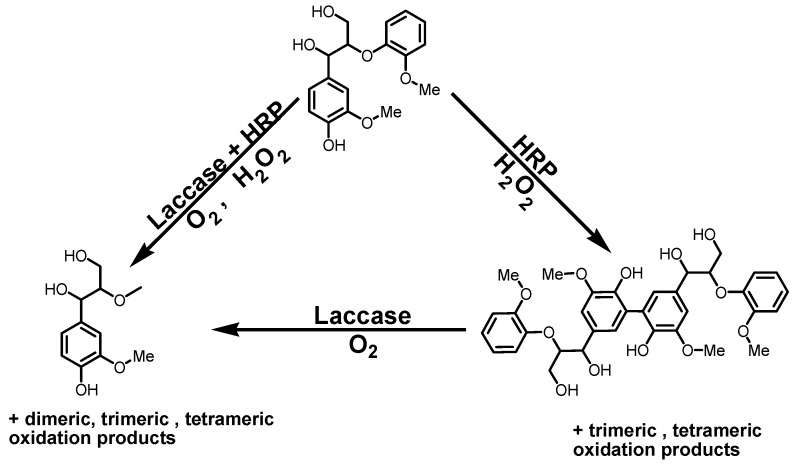
Degradation events of the lignin model compound guaiacylglycerol-β-guaiacyl ether (GGE) by multienzymatic reactions.

Recent interest in enzymatic methods for lignin biodegradation has focused on using enzymes such as peroxidases, laccase, phenol oxidases, etc. [28,29,30,31]. Peroxidases and laccases exhibit low substrate specificity and relatively wide pH of action and considered as a versatile tool towards oxidative processes [4,32,33,34,35,36,37,38]. However, the exploitation of their potentiality is limited, especially in the case of peroxidases, by their lower stability under harsh operative conditions such as high temperature, presence of surfactant, organic media and elevated level of hydrogen peroxide. Although there are quite a few reports on the use of peroxidase for the lignin oxidative process [39], horseradish peroxidase (HRP) has been shown to catalyze spontaneous polymerization of a variety of aromatic compounds [40,41]. Such undesired polymeric by-products have to be filtered out of the reactor solution in order to avoid negative feedback to the biocatalysts. On the other hand, in the case of membrane bioreactors, such polymeric products need to be avoided to circumvent clogging and fouling of the membrane. One such technique is to use a multienzyme system to convert such poly-/oligo-meric products formed by peroxidases into simpler ones. We report here a composite membrane with horseradish peroxidase (HRP) and laccase immobilized on it via the LbL assembly technique and its performance towards the degradation of a lignin model compound. The hypothesis is that by the use of such a multienzyme immobilized membrane system, any unnecessary by-products can simultaneously be converted to smaller molecules, thereby prohibiting membrane fouling, as well as enzyme inhibition. In essence, the HRP enzyme partially degrades/modifies the substrate (guaiacylglycerol-β-guaiacyl ether) to an oligomeric unit, which is then degraded by laccase to monomeric units (Scheme 1). As peroxidase enzyme needs hydrogen peroxide as one of the substrates, glucose oxidase (GO) was incorporated as a third enzyme for the in situ generation of hydrogen peroxide. Alternatively for membranes with only laccase and HRP on them, hydrogen peroxide was added to the feed. The main aim of the current study is to understand the activity of such multienzyme-functionalized membranes towards degradation of an aromatic phenolic lignin model compound, specifically guaiacylglycerol-β-guaiacyl ether (GGE). Within that context, our specific goals are: (i) fabrication and characterization of the functionalized membrane by alternating the attachment of cationic and anionic polyelectrolytes via the LbL assembly technique; (ii) investigation of the activity of the membrane reactors towards the degradation of a lignin model under convective flow conditions and analysis of the degradation products; and (iii) evaluation of the long-term performance of the enzyme-functionalized membranes.

## 2. Materials and Methods

All chemicals used during the laboratory-scale membrane fabrication and other studies were reagent grade and used without further purification. Acrylic acid (AA, 99%), potassium persulfate (KPS, >98%), guaiacylglycerol-β-guaiacyl ether (GGE) and the enzymes glucose oxidase (GO), horseradish peroxidase (HRP) and laccase from *Trametes versicolor* (powder, light brown, ≥0.5 U/mg) were purchased from Sigma Aldrich (St. Louis, MO, USA). Poly(allylamine-hydrochloride) (PAH) and *N*,*N*′-methylenebisacrylamide (MBA, >99%) were obtained from Acros, New Jersey, NJ, USA. Bradford reagent was purchased from VWR Life Science (Solon, OH, USA).

The PVDF microfiltration membranes used for all the experiments were supplied from Nanostone/Sepro Membranes Inc., Oceanside, CA, USA (PV700, pore diameter 200–450 nm, thickness of ~125 μm).

PVDF membranes were functionalized via an in situ polymerization method. Specifically, membrane functionalization was achieved via free radical polymerization of the AA to PAA (polyacrylic acid) at the PVDF pores. A monomer solution (40 mL) of AA (10 wt % aqueous solution) with MBA as the cross-linker (1.0 mol % of AA) and KPS (1.0 mol % of AA) was passed through the pores of the membrane by convective flow. The membranes were then baked at 70 °C for 1 h under N_2_ atmosphere, then washed thoroughly with DIUF (deionized ultra-filtered) water to eliminate any unreacted constituents, dried and then weighed to confirm polymerization through the mass gain. The functionalization was further confirmed through conventional spectroscopic and microscopic (SEM) analyses, as well as based on their water permeability as described below.

Attenuated total reflectance Fourier transform infrared (ATR-FTIR) spectroscopy (Varian 7000e, Palo Alto, CA, USA) was used to assess the presence of functional groups in the functionalized PVDF membranes. The samples were placed on a diamond crystal, and the spectrum was obtained between 500 and 4000 cm^−1^, averaging 24 scans at a resolution of 4 cm^−1^. The surface morphology of functionalized membranes was characterized via scanning electron microscopy (SEM, Hitachi S4300, Tokyo, Japan) and FIB-SEM (Helios Nanolab 660 from FEI, Hillsboro, USA). Membrane porosity was then estimated as the ratio of pore area to total membrane area based on SEM images of the membrane top surface. The chemical composition of the functionalized membrane was studied with X-ray photoelectron spectroscopy (K-Alpha, Thermo Fisher Scientific, West Sussex, UK).

The water permeability behavior of the PAA-functionalized PVDF membranes was studied using a laboratory-scale stainless steel pressure cell (Sepa ST, GE Osmonics, effective membrane area is 13.2 cm^2^) in dead-end mode. For pH-responsiveness studies, DIUF water at different pHs was passed through the membrane until the permeate pH was identical to the feed pH.

### 2.1. Immobilization of Enzymes in Functionalized Membranes

Enzymes were immobilized into the pores of the PAA-functionalized PVDF membrane by the layer-by-layer assembly technique. PAA having a pKa value of ~4.5 remains negatively charged after treatment with DIUF water (pH 6). In order to embed a positively-charged electrolyte into the membrane pores, 100 mL of a 45-µM solution of PAH (in DIUF water) were permeated though the membranes at pH 3.9. The electrostatic interaction between the negatively-charged carboxylic acid groups of PAA and the positively-charged ammonium groups of PAH holds the PAH immobilized in the pores. After the formation of the PAA-PAH layers, laccase was immobilized electrostatically by permeating 100 mL of a 100-ppm (10 mg/100 mL) laccase solution in DIUF water at pH 6. After embedding the laccase layer, the solution of PAH (in DIUF water) was permeated though the membranes at pH 3.9 one more time, followed by permeating HRP solution (10 mg/100 mL) in DIUF water at pH 6. This results in a membrane reactor with the pores filled up by entrapment of the mixture of enzymes around the polymer nets. The process was then repeated to have the third enzyme (GO) immobilized as the final layer. The amount of enzyme immobilized was quantified spectrophotometrically by Bradford reagent determined by the difference in the amount of the enzyme between the feed solution and the permeate. The membranes were rigorously washed with DIUF water after each immobilization step in order to wash away any loosely-bound enzymes.

The enzyme immobilized membranes were stored at 4 °C for several weeks, and the activities of these membranes towards degradation of the GGE were assessed to test their stability over a longer period.

### 2.2. Performance of the Enzyme-Immobilized Membranes towards Degradation of the Lignin Model Compound

GGE degradation studies were performed under pressure-driven flow using a laboratory-scale stainless steel pressure cell housing the functionalized membrane. In a typical degradation experiment, 100 mL of 3.1 mM solution of GGE in DIUF water were allowed to pass through the membrane, and the flow rate was varied under positive pressure using an air gas tank. The collected permeate was then analyzed for the concentrations of GGE using high-performance liquid chromatography (HPLC) with a Shimadzu instrument employing two pumps, an autosampler and a photodiode array detector. Standard solutions of GGE were prepared to obtain a standard curve which was used to quantify GGE degraded upon passage through the membrane. HPLC and LCMS were similarly used to assess the oxidation products formed. The elution was performed by pumping acetonitrile and water (40:60 *v*/*v*) at a flow rate of 0.6 mL/min. The conversion of GGE degraded by the functionalized membrane is defined here as: (C_feed_ − C_permeate_)/C_feed_.

## 3. Results and Discussions

### 3.1. Characterizations of the Functionalized PVDF Membranes

The functionalized membranes were characterized by ATR FT-IR, FIB-SEM, XPS and water permeability studies to assess the success of polymerization of PAA in the pores of PVDF membranes.

**Figure 1:**
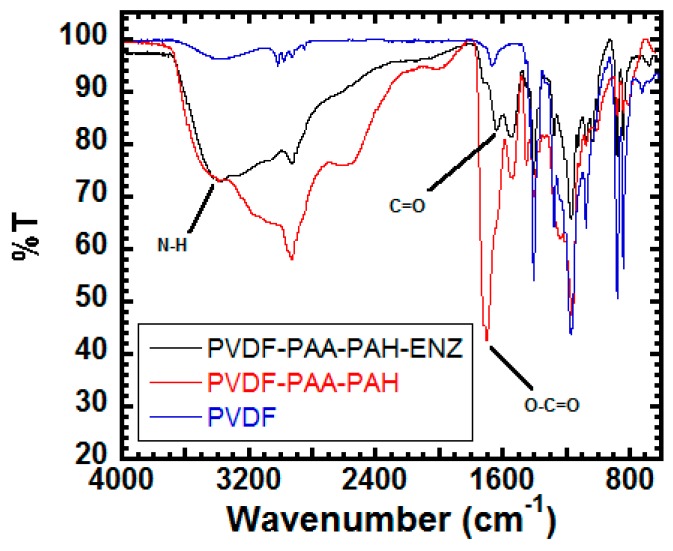
Comparison of ATR FT-IR spectra of PVDF, PVDF-PAA-PAH and PVDF-PAA-PAH-ENZ membranes.

ATR FT-IR spectroscopy was used for initial verification of successful fabrication of the PVDF and functionalized PVDF membranes. Figure 1 compares the spectra of a PVDF membrane (PVDF) as supplied, a PAA-PAH-functionalized PVDF membrane (PVDF-PAA-PAH) and an enzyme-functionalized PVDF membrane (PVDF-PAA-PAH-ENZ). The characteristic absorption peaks of the CF_2_ groups of the PVDF chains lie in the region of 1050–1280 cm^−1^ for all the membranes [42]. The appearance of new peaks at 1720 cm^−1^ and 1544 cm^−1^ (Figure 1, red line) corresponding to the carbonyl stretch and anti-symmetric stretching of carboxyl groups (COOH), respectively, verify successful polymerization of acrylic acid in the membrane matrix [43]. Enzymes usually have two absorption bands, near 1645 cm^−1^ and 1540 cm^−1^, corresponding to the peptide backbone amide I and amide II modes, respectively [44]. Our enzyme-functionalized PVDF membranes showed similar peaks (Figure 1, black line). The peak at 1642 cm^−1^ was due to the stretching vibration of the C=O amide bond, and the 1545 cm^−1^ peak was assigned to the combination of the bending vibration of the N-H bond and the stretching vibration of the C–N bond of the peptide backbone, signifying immobilization of enzymes on the membrane support [35]. The peak around 2920 cm^−1^ and 2850 cm^−1^, in all the samples, was attributed to the asymmetric and symmetric C–H stretch. The band peaks in the range of 3500–3300 cm^−1^ were associated with a combination of the N–H stretch and O–H stretch belonging to the protein and the membrane matrix.

**Figure 2:**
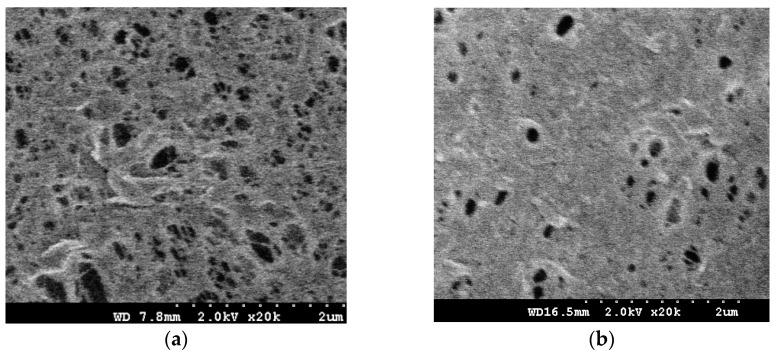

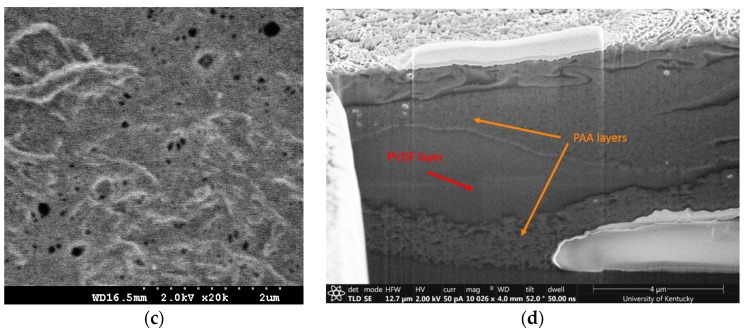
Scanning electron microscopy (SEM) images of (**a**) bare PVDF membrane, (**b**) PAA-functionalized PVDF membrane (PVDF-PAA) and (**c**) enzyme immobilized PVDF membrane (PVDF-PAA-PAH-ENZ) and (**d**) FIB-SEM image of the inside morphology of the pores of the functionalized membrane (PVDF-PAA).

The surface morphologies of the PVDF and functionalized PVDF membranes were characterized by scanning electron microscopy (SEM). FIB (focused-ion-beam) SEM was used to access the inside of the fabricated membrane pores as shown in Figure 2. The bare PVDF membranes (Figure 2a) are fairly porous with an average pore size of 225 ± 50 nm. After the PAA polymerization, the morphology of the membrane pores changed substantially, shrinking the pores to an average size of 100 ± 5 nm (Figure 2b). Successive modification of the membrane after immobilizing PAH and the enzymes shrunk the pores further, reducing the average diameter to 70 ± 12 nm, as can be seen in Figure 2c. Figure 2d shows the inside morphology of the pores of the functionalized membrane using FIB-SEM. The distinction between the PVDF background and the polymeric layer immobilized on it is very clear from the FIB-SEM; however, it is hard to distinguish between an enzyme layer and a PAA-polymeric layer.

**Figure 3:**
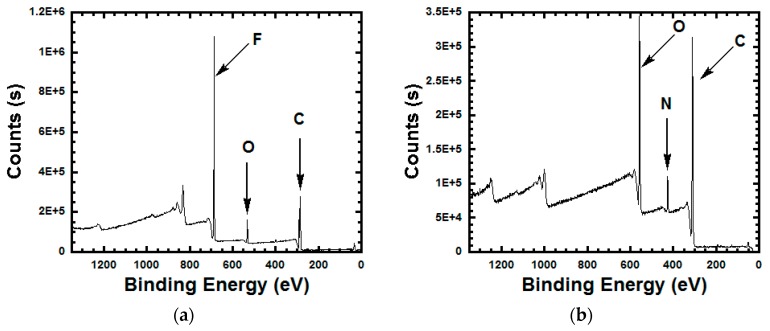

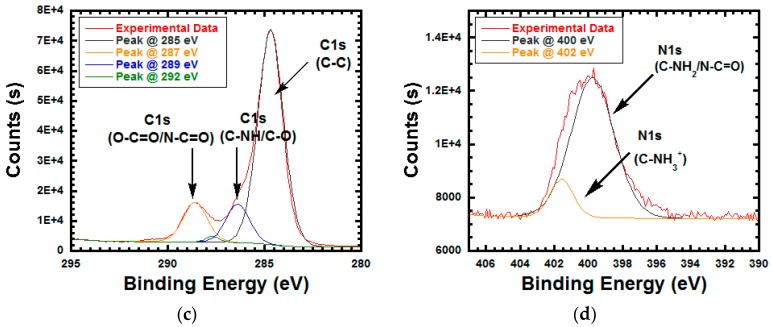
XPS survey spectra of (**a**) bare PVDF and (**b**) functionalized PVDF membranes (PVDF-PAA-PAH-ENZ). (**c**) C1s core-level spectra and (**d**) N1s core-level spectra of the functionalized PVDF membrane.

The surface compositions of bare PVDF membranes and enzyme-functionalized PVDF membranes (PVDF-PAA-PAH-ENZ) were explored by X-ray photoelectron spectroscopy (XPS). Survey spectra of these membranes exhibit significant N and considerably higher C content in the functionalized PVDF membrane (Figure 3b) compared to the bare PVDF membrane (Figure 3a). The N content in the functionalized membranes is due to the presence of the polyallylamine, as well as from the peptide backbone of the enzymes, consistent with our earlier findings [45]. Deconvolution of the C1s peak near 290 eV for the functionalized membrane yielded three peaks corresponding to carbon in C–C (285 eV), C–O (287 eV) and the C=O (289 eV) functionalities (Figure 3c), in addition to a signature of C–F attributed to the PVDF support [45,46]. Deconvolution of the N1s core-level spectra of the functionalized membrane resulted in two peaks at 399 eV and 401 eV, and these were assigned to C–N (amine/amide) from the enzyme and ammonium salt (C–NH_3_^+^) from the polyallylamine layer, respectively.

**Figure 4:**
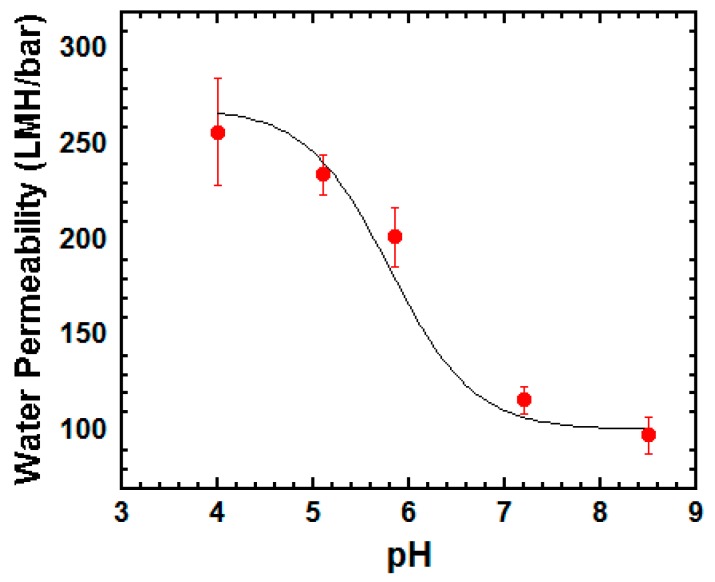
Effect of pH on the water permeability of PVDF-PAA membranes. PAA content = 10 wt %. PVDF-PAA membrane area = 13.2 cm^2^. T = 22 °C. Cross-linker (*N*,*N*′-methylenebisacrylamide (MBA)) concentration of 1 mol% of monomer, acrylic acid (AA).

Finally we profiled the effect of pH on the water permeability of PVDF-PAA-functionalized membranes (Figure 4), which serves as a definitive test of PAA functionalization inside the membrane pores [47,48]. The flux of the membrane decreased with increasing pH due to the expected swelling of the PAA hydrogel inside the pores [49]. The water permeability data were fit with Equation (S1) [50] yielding a pKa of 5.6.

### 3.2. Reactivity of the Membrane Bioreactors towards GGE Degradation/Modification

**Figure 5:**
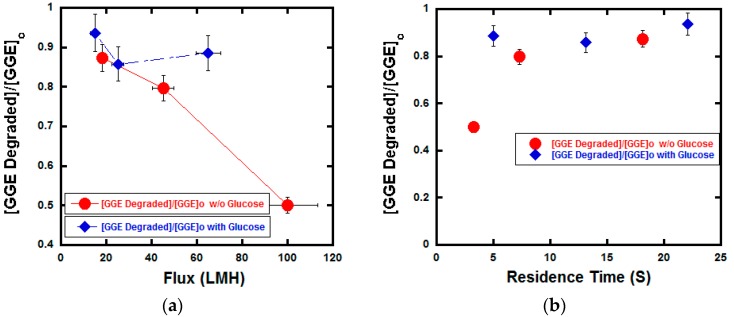

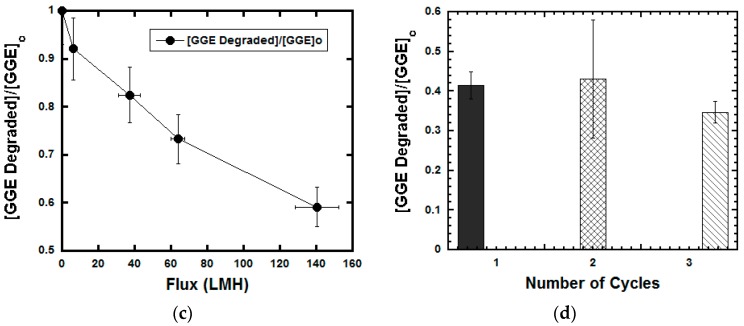
Degradation of GGE (**a**) by the multienzyme-functionalized membrane, (**b**) as a function of residence time, (**c**) by the laccase-functionalized membrane as a function of flow rate and (**d**) by the activity of the multienzyme-functionalized membrane towards GGE degradation after multiple cycles. Experiments were performed at a temperature of 22 °C and a pH of 5.6. The initial substrate concentration was 3.1 mM GGE in water. LMH, L·m^−2^·h^−1^.

The multienzyme immobilized membranes were fabricated as described in the Materials and Methods Section. The initial activity of the laccase and HRP enzymes were measured in the solution state prior to immobilization by the conventional colorimetric assay in the presence of 2,2´-azinobis(3-ethylbenzothiazoline-6-sulfonic acid)-diammonium salt (ABTS) (please see Supporting Information Figure S7). It should be noted here that the presence of multiple enzymes in the membrane makes it difficult to measure the individual activity of the immobilized enzymes. However, the loading of the enzyme into the membrane matrix was confirmed by the Bradford protein assay of the enzyme feed and the permeate during enzyme immobilization. In general, 35–60% of each enzyme could be loaded on the membranes. The applicability of the enzyme-functionalized membrane towards degradation of the lignin model compound was demonstrated by passing an aqueous solution of GGE through the membrane in a dead-end cell. The multienzyme immobilized membranes had three enzymes on them (Lac, HRP and GO) to be able to work as a bioreactor. It should be noted here that laccase uses oxygen, whereas the enzyme HRP uses hydrogen peroxide as the electron accepting secondary substrate during the oxidation of the primary substrate. A continuous oxygen atmosphere for the laccase enzyme was maintained by air flow from the air gas tank, which also maintained necessary positive pressure for controlling the flow rate. During the experiments the necessary concentration of hydrogen peroxide was maintained by adding glucose in to the feed, which upon reaction with GO produces hydrogen peroxide in situ. HPLC and LC-MS analyses were used to monitor the diminution of the GGE content in the permeate, as well as to detect the presence of various oxidation products of GGE (please see Supporting Information, Figure S2). Figure 5a portrays the degradation of GGE by enzymatic membranes as a function of different flow rates. The data in Figure 5a were from the Lac-HRP-GO membrane (Feed 3.1 mM GGE) in the presence (blue diamonds) or absence (red circles) of glucose (3 mM) in the feed. The approximate amount of enzymes on the membrane used in this experiment were laccase 5.6 mg, peroxidase 5.3 mg and glucose oxidase 3.9 mg. The data indicate that with glucose in the feed, close to 95% of the GGE could be degraded at a flow rate of 15 L·m^−2^·h^−1^ (LMH) under an applied pressure of around 4 bars. At such a slow flow rate (high residence time, 22 s; Figure 5b), comparable degradation could also be achieved without glucose in solution (~85%, 17-s residence time). Comparison at a higher flow rate (lower residence time) reveals that the membrane worked much better when all three enzymes work simultaneously. A degradation as high as 90% was achieved at 64 LMH (5s residence time, Figure 5b) with glucose in the feed. In contrast, in the absence of glucose in the feed, the membrane could degrade only 65% of the initial GGE under a similar flow rate. The data in Figure 5c are the GGE degradation profile from an independent laccase immobilized membrane. These data closely resemble the data from the multienzyme membrane reactor without glucose in the feed (Figure 5a, red circle) to prove that in the absence of the necessary substrate, hydrogen peroxide, the multienzyme membrane reactor could act as a single enzyme membrane reactor. This demonstrates the efficacy of the multienzyme membrane towards the degradation of lignin model compounds. Figure 5d shows the reusability (three repeated cycles) of the multienzyme-functionalized membrane as assessed by retention of its capacity to catalyze the conversion of GGE under convective flow mode. In this case, a membrane with laccase (3.3 mg) peroxidase (2.4 mg) was used, and a stoichiometric amount of hydrogen peroxide was added into the feed (3.1 mM of GGE in water). Each cycle was comprised of passing 100 milliliters of feed over five hours of operation. As evident from the Figure 5d, our enzyme-functionalized membrane displayed encouraging retention of activity, losing only 12% of its initial activity after multiple cycles of operation. This is consistent with our earlier observation [45], where we have discussed the similar stability of the enzyme immobilized membrane matrix.

Effort was made to characterize the degraded products from the enzymatic reactions. LC-MS analysis of the reaction products of GGE conversion revealed multiple oxidized products with *m*/*z* ranging from 251 [M + Na]^+^ up to 979 [M + Na]^+^. Enzymatic conversion of GGE (MW 320) to multiple polymeric oxidized products with higher molecular weight has been reported by other researchers, as well [51]. It is noteworthy to point out that the generation of such oligomeric products is enzyme dependent. Our findings on this are that horseradish peroxidase generated the high molecular weight dimeric (*m*/*z* 661, **B**) and trimeric (*m*/*z* 979, **C**) products to a greater extent than the low molecular weight (*m*/*z* 251, **A**). However, laccase produced a higher amount of **A** (*m*/*z* 251) than **B** (*m*/*z* 661), and none of the higher ones were seen. To be specific, laccase produced 99% of **A** and only 0.8% of **B** of the total GGE conversion product. In contrast, with HRP only 43% of the total GGE conversion product was **A**. The formation of **B**, in this case, was ten-times more than that produced from the laccase reaction. Moreover, about 2% of the trimeric adduct **C** was formed with HRP, which was not seen in the laccase reaction. All the data are tabulated in Table 1. The effect was also seen in the case of the multienzyme membrane with the formation of 62% of the degradation product **A** compared to only 0.7% and 0.4% of the oligomeric products **B** and **C** respectively. Based on GGE degradation patterns, it can be concluded that reactions performed by HRP resulted in oxidative oligomerization, probably formed through C–C coupling of the phenolic units [52]. Laccase, on the other hand, degraded the GGE and the oligomeric products from the HRP reactions through an alkyl-phenyl ether bond cleavage reaction [38]. The fact that laccase was able to break down such oligomers to monomeric units justifies the unification of multiple enzymes with the membrane reactor to protect its activity. Various such multienzymatic systems have been studied from time to time [7,8,9,39,53]. While immobilized enzymes generally have better stability over the solution phase, the shortening of the diffusion time of the substrate or transformed substrate from one enzyme to another enzyme in multienzymatic systems makes them more potent with higher observed catalytic activity. Jia et al. recently discussed a comparison of the efficiency of substrate conversion by such a multienzymatic system to free enzyme, and in a few cases, a decrease in performance was also observed [8].

## 4. Conclusions

Transformation of lignin macromolecules to value-added small molecules through enzymatic degradation on a membrane platform was the primary objective of this study. Successful fabrication of multienzyme (laccase, peroxidase and glucose oxidase) immobilized PVDF microfiltration membrane bioreactors was demonstrated. The functionalized membranes were used for oxidative degradation of the lignin model compound GGE. Our multienzyme immobilized membranes, engineered through the layer-by-layer assembly method, were capable of breaking the main chain linkage in lignin-type molecules. Preliminary investigation revealed over 90% of initial GGE degradation with the multienzyme immobilized membranes under the optimum flow rate. A combination of HPLC, LC-MS analysis on the GGE conversion product confirmed the formation of oligomeric oxidation products upon reaction with peroxidase. The laccase enzymes present in the bioreactors were able to further degrade these oligomeric units, validating the potential use of multienzyme membrane systems. Retention of enzymatic activity (towards GGE degradation) of the membrane reactors was established up to multiple cycles of repetitive use. The membrane reactors were active for about a month time of storage at 4 °C. This study opens up perspectives for further enhancement of multienzyme membrane reactor systems and indicates their potential for applications in the field of the biodegradation of renewables.

## Figures and Tables

**Table 1:** Conversion of GGE to different oxidation products.

Reactors	Of the Total GGE Conversion Product			Total GGE Conversion
	A (*m/z* 251)	B (*m/z* 661)	C (*m/z* 979)	
Laccase	99%	0.8%	-	73%
HRP	43%	9.9%	2.4%	57%
Multienzyme	62%	0.7%	0.4%	95%

## Supplementary Materials

The following are available online at http://www.mdpi.com/2073-4360/10/4/463/s1: Figure S1: Comparison of the degradation of GGE with laccase and multienzyme immobilized membranes in flow-through experiments as studied by UV-Vis spectroscopy. Experiments were performed at a temperature of 22 °C and a pH of 5.6, Figure S2: Degradation of GGE (initial GGE concentration 3.1 mM) with the PVDF-PAA-PAH-ENZ membrane in a flow-through experiment as studied by HPLC, Figure S3: Plot of GGE concentration (initial GGE concentration 3.1 mM) as passed through the PVDF-PAA-PAH membrane in a flow-through experiment as studied by HPLC. This is to show that with no enzyme present on the membrane, GGE could not be degraded. Furthermore, only a minimal (~5%) or no absorption of GGE onto the membrane matrix was observed, Figure S4: Mass spectrum of the GGE permeate degraded by a laccase immobilized membrane, Figure S5: Mass spectrum of the GGE permeate degraded by an HRP immobilized membrane, Figure S6: Mass spectrum of the GGE permeate degraded by a multienzyme immobilized membrane, Figure S7: Solution phase activity of (a) laccase and (b) HRP used for immobilization.
