# Supplementary material for: Multienzyme Immobilized Polymeric Membrane Reactor for the Transformation of a Lignin Model Compound
Source: Polymers (Basel). 2018 Apr 23;10(4):463. doi: 10.3390/polym10040463 (PMC6358281; doi:10.3390/polym10040463)
Supplement: Supplementary file 1 [file polymers-10-00463-s001.pdf]

Supporting Information: Multienzyme Immobilized Polymeric Membrane Reactor for Transformation of Lignin Model Compound

Rupam Sarma, Md. Saiful Islam, Mark P. Running and Dibakar Bhattacharyya

The equation used to fit the water permeability data at various pH:

$$Lp = \left\{ L_{p,max}^{\frac{1}{2}} - \frac{[COO^-]}{[COOH] + [COO^-]} \left(L_{p,max}^{\frac{1}{2}} - L_{p,min}^{\frac{1}{2}} \right) \right\}^2 \qquad \text{(S1)}$$

where

$$\frac{[COO^-]}{[COOH] + [COO^-]} = \frac{1}{1 + 10^{(pKa - pH)}}$$

Here $L_{p,max}$ = 257.4 and $L_{p,min}$ = 98.2, where L_p is membrane water permeability, LMH/bar.

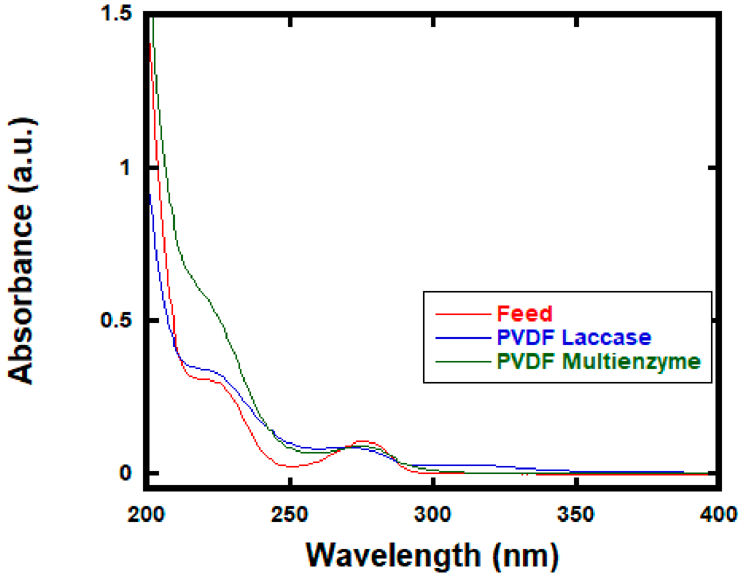

Figure S1. Comparison of degradation of GGE with laccase and multienzyme immobilized membranes in flow through experiments as studied by UV-Vis Spectroscopy. Experiments were performed at a temperature of 22 °C and a pH of 5.6.

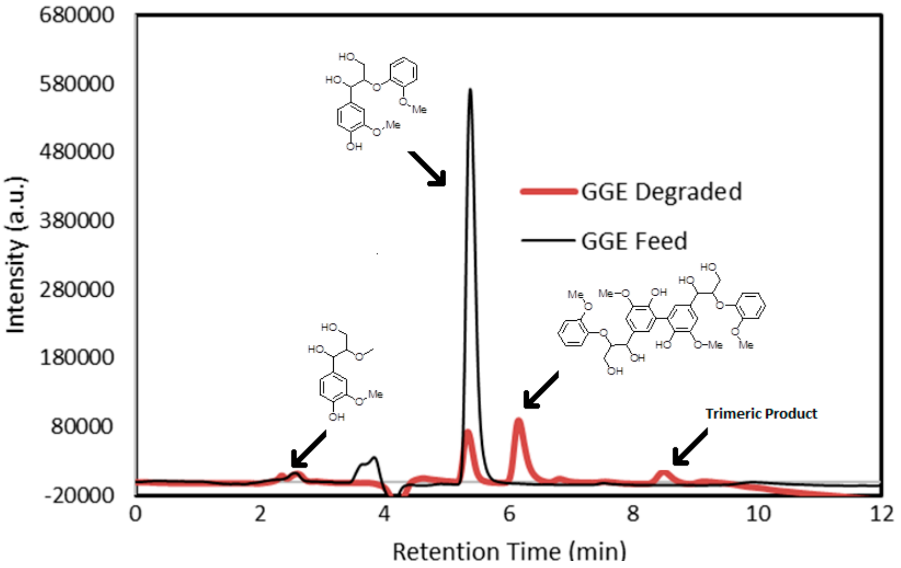

Figure S2. Degradation of GGE (initial GGE Concentration 3.1 mM) with PVDF-PAA-PAH-ENZ membrane in a flow through experiment as studied by HPLC.

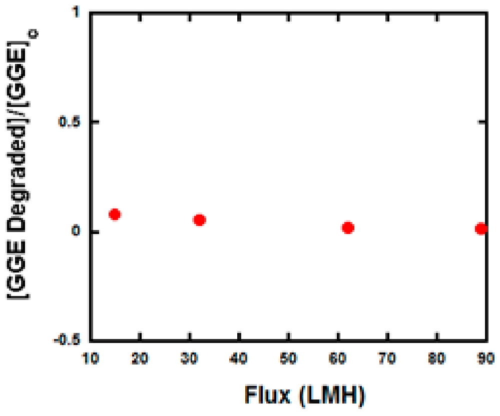

(a) **(b)**

Figure S3. Plot of GGE concentration (initial GGE Concentration 3.1 mM) as passed through PVDF-PAA-PAH membrane in a flow through experiment as studied by HPLC. This is to show that with no enzyme present on the membrane GGE could not be degraded. Also, only a minimal (~5%) or no absorption of GGE onto the membrane matrix was observed.

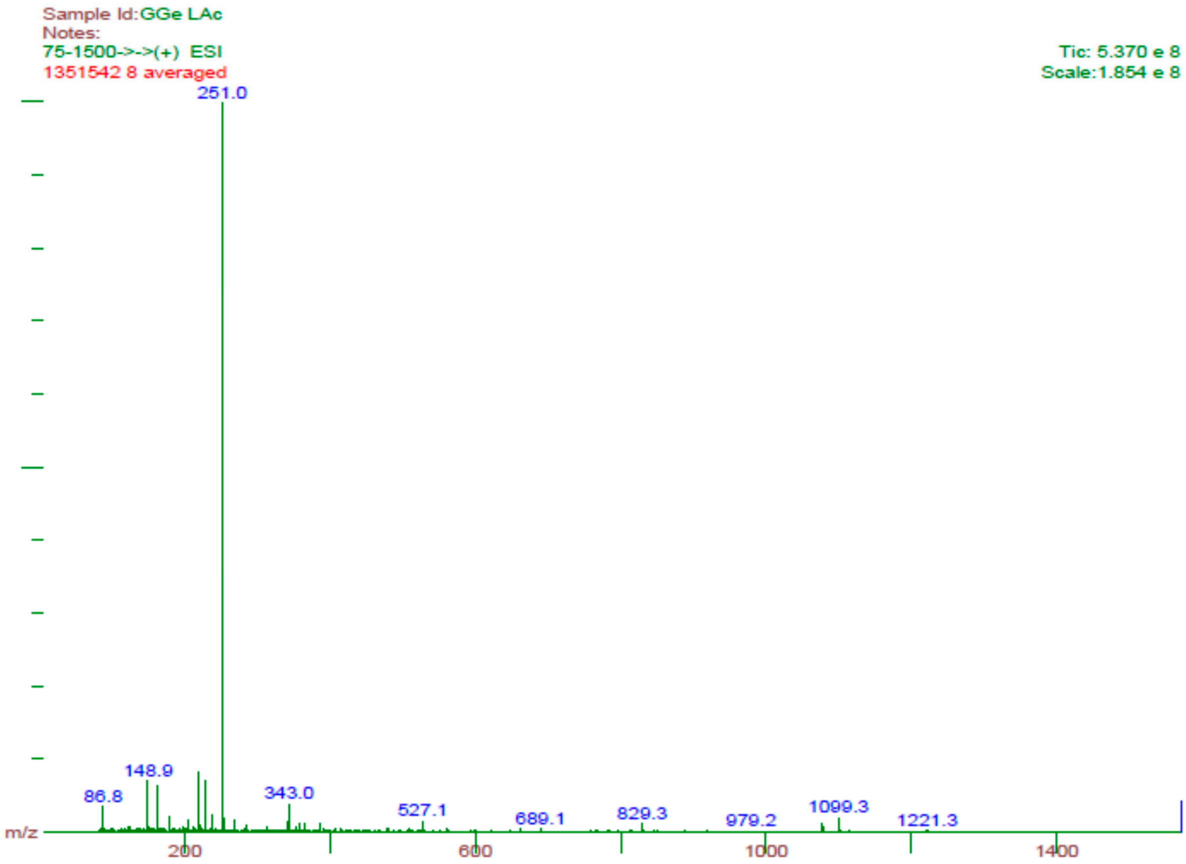

Figure S4. Mass Spectrum of GGE permeate degraded by a laccase immobilized membrane.

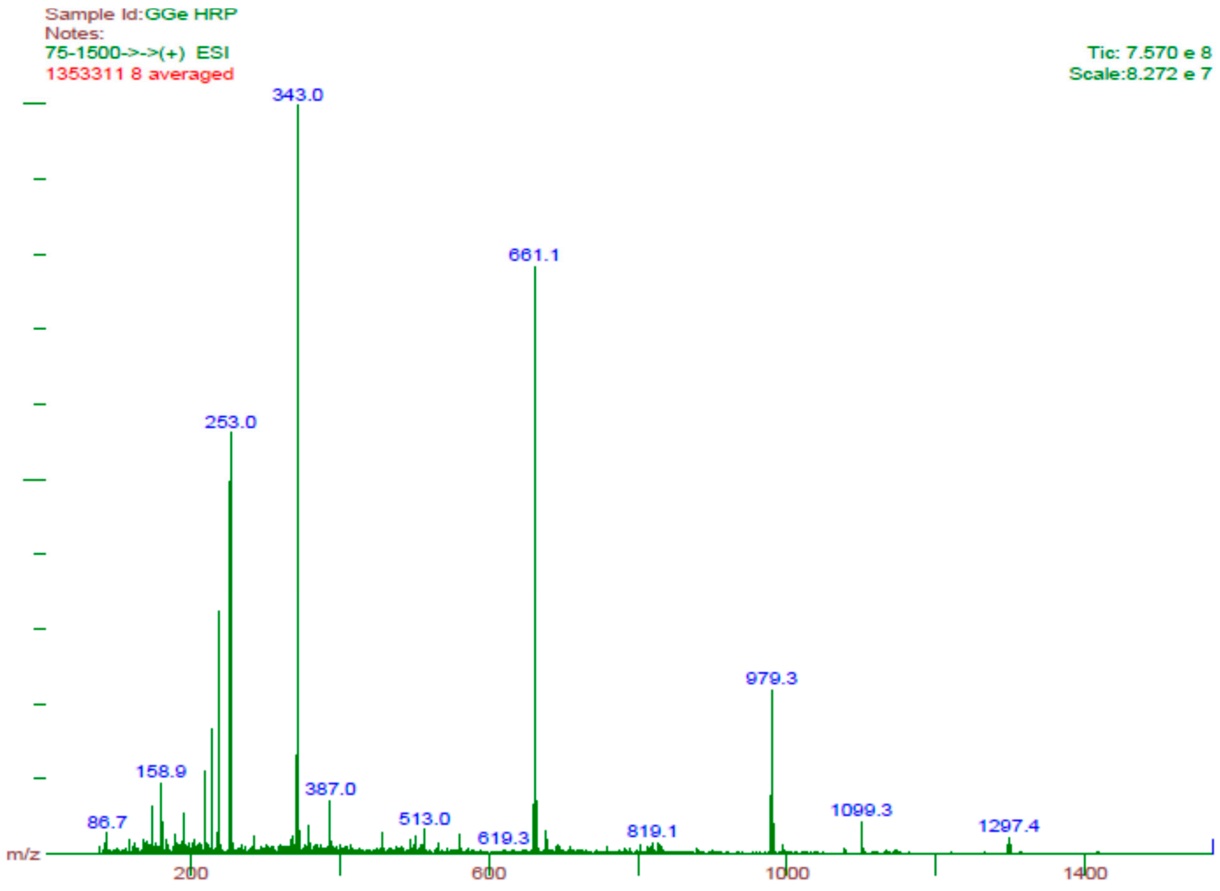

Figure S5. Mass Spectrum of GGE permeate degraded by a HRP immobilized membrane.

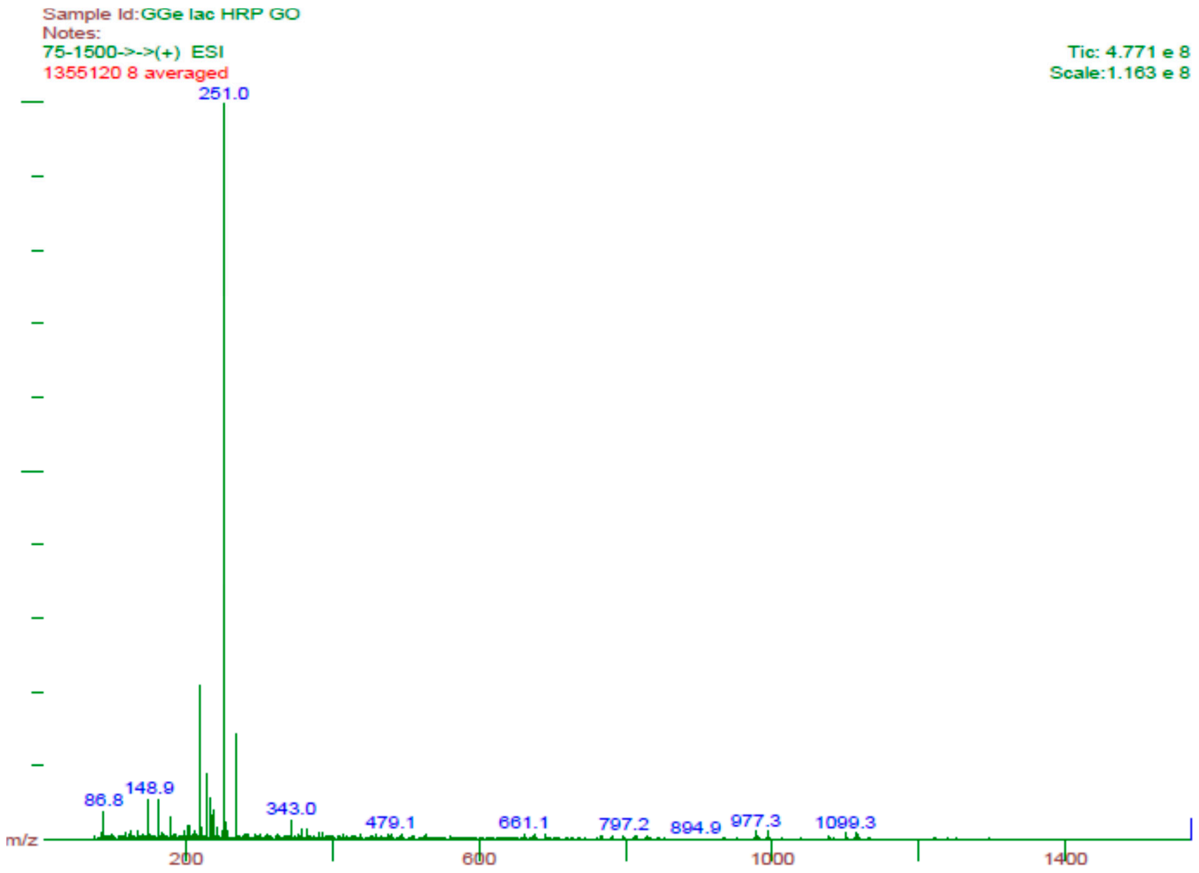

Figure S6. Mass Spectrum of GGE permeate degraded by a multienzyme immobilized membrane.

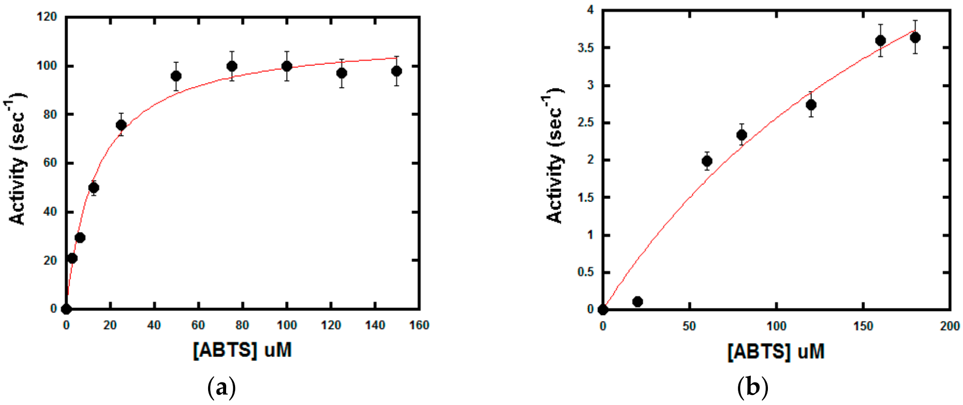

Figure S7. Solution phase activity of (**a**) laccase and (**b**) HRP used for immobilization.
